# Burden of *Clostridium difficile-*associated disease among patients residing in nursing homes: a population-based cohort study

**DOI:** 10.1186/s12877-016-0367-2

**Published:** 2016-11-25

**Authors:** Holly Yu, Onur Baser, Li Wang

**Affiliations:** 1 Pfizer Inc, Arcola Road, Collegeville, PA 19426 USA; 2 Center for Innovation & Outcomes Research, Department of Surgery, Columbia University, New York, NY USA; 3 STATinMED Research, New York, NY USA; 4 STATinMED Research, Plano, TX USA

**Keywords:** *Clostridium difficile* (*C. difficile*), CDI, Nursing homes, Medicare, Medicaid

## Abstract

**Background:**

*Clostridium difficile* (*C. difficile*) infection (CDI) is the leading cause of nosocomial diarrhea in the United States. This study aimed to examine the incidence of CDI and evaluate mortality and economic burden of CDI in an elderly population who reside in nursing homes (NHs).

**Methods:**

This was a population-based retrospective cohort study focusing on US NHs by linking Medicare 5% sample, Medicaid, Minimum Data Set (MDS) (2008–10). NH residents aged ≥65 years with continuous enrollment in Medicare and/or Medicaid Fee-for-Service plan for ≥12 months and ≥2 quarterly MDS assessments were eligible for the study. The incidence rate was calculated as the number of CDI episodes by 100,000 person-years. A 1:4 propensity score matched sample of cohorts with and without CDI was generated to assess mortality and health care costs following the first CDI.

**Results:**

Among 32,807 NH residents, 941 residents had ≥1 episode of CDI in 2009, with an incidence of 3359.9 per 100,000 person-years. About 30% CDI episodes occurred in the hospital setting. NH residents with CDI (vs without CDI) were more likely to have congestive heart failure, renal disease, cerebrovascular disease, hospitalizations, and outpatient antibiotic use. During the follow-up period, the 30-day (14.7% vs 4.3%, *P* < 0.001), 60-day (22.7% vs 7.5%, *P* < 0.001), 6-month (36.3% vs 18.3%, *P* < 0.001), and 1-year mortality rates (48.2% vs 31.1%, *P* < 0.001) were significantly higher among the CDI residents vs non-CDI residents. Total health care costs within 2 months following the first CDI episode were also significantly higher for CDI residents ($28,621 vs $13,644, *P* < 0.001).

**Conclusions:**

CDI presents a serious public health issue in NHs. Mortality, health care utilization, and associated costs were significant following incident CDI episodes.

**Electronic supplementary material:**

The online version of this article (doi:10.1186/s12877-016-0367-2) contains supplementary material, which is available to authorized users.

## Background

*Clostridium difficile* (*C. difficile*) infection (CDI) is the leading cause of nosocomial diarrhea in the United States and has surpassed the infection rate of other health care-associated infections (HAI) such as methicillin-resistant *Staphylococcus aureus* [1, 2]*.* In a recent national prevalence survey, *C. difficile* was the most commonly-reported pathogen, accounting for 12% of HAIs and an estimated 80,400 hospital-onset infections [3]. *C. difficile* was responsible for almost a half-million infections and approximately 28,000 deaths in 2011 [4]. The emergence of the epidemic BI/NAP1/027 strain has been associated with the increased incidence, severity, and mortality of CDI [5]. The increasing incidence of CDI also imposes a large financial burden on the health care system [6]. In 2008, the excess health care costs for CDI in acute care facilities alone were approximately $4.8 billion [7].

The elderly represent the population at greatest risk for CDI, accounting for the highest disease morbidity and mortality. Moreover, residents of nursing homes and other long-term care (LTC) facilities are more prone to CDI due to advanced age, frequent hospitalization, prevalent comorbid illnesses, extended stays, and frequent exposure to antibiotics [1]. In 2013, Garg et al. determined that there was a predominantly higher number of patients diagnosed with LTC-acquired CDI (46.1%) compared to community-acquired (33.3%) or hospital-acquired (20.6%) CDI [8]. Additionally, statewide surveillance in Ohio has found that there were more cases of CDI diagnosed in LTC facilities than in acute-care settings [9]. The increasing trends of CDI-incidence in LTC facilities such as nursing homes highlight the need for optimal *C. difficile* infection control measures [8].

Previous studies have focused on the costs related to CDI in acute-care facilities [10, 11]. However, to our knowledge, there are no recent studies assessing the costs of CDI in nursing homes. Therefore, the objectives of this study were to examine the incidence of CDI and evaluate the mortality and economic burden of CDI in an elderly population residing in nursing homes.

## Methods

### Study design and data sources

This population-based retrospective cohort study included adults aged ≥65 years enrolled in Medicare or with dual eligibility with the Medicaid fee-for-service (FFS) health plan during 2008–2010. A Medicare 5% random sample was used, and all eligible enrollees were required to have both Part A (hospital insurance) and Part B (supplementary medical insurance) during the study period [12]. Because some Medicare enrollees, may also be covered by Medicaid due to socioeconomic factors, we linked the Medicare 5% sample with the 100% Medicaid claims data.

To identify Medicare enrollees residing in nursing homes, we linked the 5% Medicare sample and 100% Medicaid with the 100% Minimum Data Set (MDS) and required ≥2 consecutive MDS quarterly assessments in the 12 months prior to the CDI diagnosis. The MDS is a federally-mandated nursing home clinical assessment performed on all residents at admission, discharge, annually, and quarterly, regardless of the payer.

### Identification of CDI episodes

The primary outcome was the incidence of CDI, identified as residents with ≥1 medical claim for CDI (International Classification of Diseases, 9th Revision, Clinical Modification [ICD-9-CM] code: 008.45) in Medicare or Medicaid data, or identified CDI (1 = yes) in MDS data from January 1, 2009 through December 31, 2009. An incident CDI episode was defined as a patient with no occurrences of CDI within 60 days prior to the first CDI claim. Among patients with multiple episodes of CDI, each episode was considered as incident if there was at least a 60-day gap between the 2 episodes. We also identified the setting (eg, inpatient, nursing home) of infection onset using the first CDI claim setting.

### Case-control analysis

As a secondary objective, we examined mortality at 30, 60, and 180 days, and at 1 year following the onset of the index CDI episode. Since mortality information is derived from the Medicare enrollment file which is updated by the Social Security Administration, we were able to capture all deaths. We also analyzed all-cause health care costs and utilization stratified by health plan (eg, Medicare and Medicaid) within 2 months following the first incident episode among those with CDI (cases) and those without CDI (controls). For this analysis, in contrast to the incidence computation, residents were required to have no diagnosis of CDI within the 12 months prior to the first incident episode (index date) in 2009. The 12 months prior to this index date was used as an observation period to allow sufficient time to determine baseline demographic and clinical characteristics and health care utilization parameters. Residents with CDI episodes identified in the MDS typically lacked complete Medicare and Medicaid data; therefore, these residents were excluded from this portion of the analysis. Eligible residents enrolled in Medicare or Medicaid without a CDI diagnosis between January 1, 2008 and December 31, 2010, served as the control group and were randomly assigned an index date in 2009.

### Statistical analyses

The incidence rates of CDI were calculated as the number of residents with a CDI episode per 100,000 person-years and stratified by age, gender, race, and region. For the case-control analysis, each CDI case resident was matched to 4 non-CDI control residents using a ‘greedy’ match method [13]; CDI cases were matched to non-CDI controls within 0.001 units of the propensity score [14]. The propensity score was calculated via a logistic regression model of factors associated with the risk of CDI, including the residents’ age, gender, race, US census region, comorbidities using the Charlson Comorbidity Index (CCI) score, prior hospitalizations, nursing home stays, antibiotic exposure, gastric acid suppressant use within 90 days and 1 year prior to the incident CDI, individual comorbidities, and comorbid conditions included the MDS data.

Chi-square tests were used to evaluate differences in categorical variables. Student *t*-tests were used for evaluating differences in continuous variables. In addition to *p*-values, standardized differences were calculated for each variable. A standardized difference of >10% was used to assess significant practical differences in the case-control comparison [15].

Statistical significance was set *a priori* at the alpha <0.05. All analyses were performed using Statistical Analysis System (SAS) version 9.3 (SAS Institute Inc., Cary, NC).

## Results

### CDI incidence rates

We identified 1,051 incident episodes of CDI among 941 residents with ≥1 CDI episode in a cohort of 32,807 residents who met the eligibility criteria. The annual incidence density of CDI was 3,752.7 episodes per 100,000 person-years and 3,359.9 residents per 100,000 person-years (Table 1). Across the age strata, an inverse linear trend was observed: the incidence ranged from a low of 3,414.9 episodes per 100,000 person-years among residents aged ≥85 years to a high of 4,247.5 episodes/ per 100,000 person-years among those aged 65–74 years. Incidence was slightly higher among men than women and lower among whites as compared to blacks or other race categories. Geographically, the incidence density of CDI was highest in the Northeast (4,641.1 per 100,000 person-years), and lowest in the Midwest (3,306.8 episodes per 100,000 person-years). The majority of CDI episodes occurred in a nursing home (71.7%), and 28.3% occurred in an inpatient hospital setting.

**Table 1:** Incidence Rate of CDI Stratified by Age, Sex, Race, and Region

	Incident CDI Cases per 100,000 Person-years of Observation	#of CDI Residents per 100,000 Person-years of Observation
Overall	3752.7	3359.9
Incidence by Age Group		
65–74	4247.5	3792.4
75–84	4048.1	3598.3
85 and older	3414.9	3075.5
Incidence by Sex		
Male	4339.3	3789.2
Female	3573.8	3229.0
Incidence by Race		
White	3614.2	3244.5
Black	4252.8	3750.9
Other	4583.6	4118.6
Incidence by Region		
Northeast	4641.1	4188.6
Midwest	3306.8	2941.6
South	3507.0	3170.4
West	3657.0	2904.1

*CDI Clostridium difficile* infection

### Case-control analysis

After accounting for the inclusion and exclusion criteria, a sample of 789 residents with an incident CDI episode and 30,258 without were included for this analysis. The mean age of the residents with and without CDI was 83.1 years and 83.6 years, respectively. Geographically, a higher proportion of CDI residents resided in the Northeast region (36.1% vs 28.5%, *P* < 0.001), whereas a higher proportion of non-CDI residents resided in the Midwest (34.3% vs 27.8%, *p* < 0.001; Table 2). Approximately two-thirds of residents had Medicaid coverage along with their Medicare insurance.

**Table 2:** Baseline Demographic Characteristics Between CDI and Non-CDI Residents Before and After PSM

	Unmatched Groups						1:4 PSM-Matched Groups					
	Non-CDI Residents		CDI Residents				Non-CDI Residents		CDI Residents			
	(*N* = 30258)		(*N* = 789)				(*N* = 2612)		(*N* = 653)			
	N/Mean	%	N/Mean	%	*P*-value	Std. Diff.	N/Mean	%	N/Mean	%	*P*-value	Std. Diff.
Age (mean ± SD)	83.6 ± 9.3		83.1 ± 8.8		0.1103	5.6	83.5 ± 9.1		83.6 ± 8.7		0.9206	0.4
65–74	5979	19.8%	156	19.8%	0.9934	0.0	501	19.2%	116	17.8%	0.4082	3.6
75–84	8541	28.2%	237	30.0%	0.2648	4.0	770	29.5%	200	30.6%	0.5657	2.5
85 or older	15738	52.0%	396	50.2%	0.3117	3.6	1341	51.3%	337	51.6%	0.9025	0.5
Gender												
Female	23020	76.1%	585	74.1%	0.2089	4.5	1946	74.5%	498	76.3%	0.3535	4.1
Region												
Northeast	8610	28.5%	285	36.1%	<0.001	16.4	895	34.3%	230	35.2%	0.6453	2.0
Midwest	10375	34.3%	219	27.8%	0.0001	14.2	739	28.3%	185	28.3%	0.9845	0.1
South	10239	33.8%	261	33.1%	0.6563	1.6	905	34.6%	224	34.3%	0.8685	0.7
West	1033	3.4%	23	2.9%	0.4453	2.8	73	2.8%	14	2.1%	0.3557	4.2
Race/Ethnicity												
White	24337	80.4%	612	77.6%	0.0455	7.0	2065	79.1%	527	80.7%	0.3523	4.1
Black	4198	13.9%	128	16.2%%	0.06	6.6	392	15.0%	92	14.1%%	0.5545	2.6
Other	1723	5.7%	49	6.2%	0.5373	2.2	155	5.9%	34	5.2%	0.4765	3.2
Insurance Type												
Medicare only	5812	19.2%	151	19.1%	0.9607	0.2	460	17.6%	129	19.8%	0.2025	5.5
Medicaid only	5221	17.3%	112	14.2%	0.0245	8.4	366	14.0%	84	12.9%	0.4463	3.4
Medicare and Medicaid dual	19225	63.5%	526	66.7%	0.0712	6.6	1786	68.4%	440	67.4%	0.6252	2.1

*CDI Clostridium difficile* infection, *SD* standard deviation; *Std. Diff* standardized difference, *PSM* propensity score matching

Residents with CDI were more often diagnosed with comorbidities than those without CDI (CCI scores: 4.6 ± 2.8 in CDI vs 3.2 ± 2.4 in non-CDI groups, *P* < 0.001; Additional file 1: Table S1). The hospitalization burden among those with CDI within the preceding 1 year (64.8% vs 32.4%, *P* < 0.001) or 90 days (43.2% vs 10.9%, *P* < 0.001) was significantly higher compared to residents without CDI. After propensity score matching (PSM), 653 residents with an incident CDI episode and 2,612 residents without a CDI episode were compared for the secondary objectives.

### Mortality

**Fig. 1:**
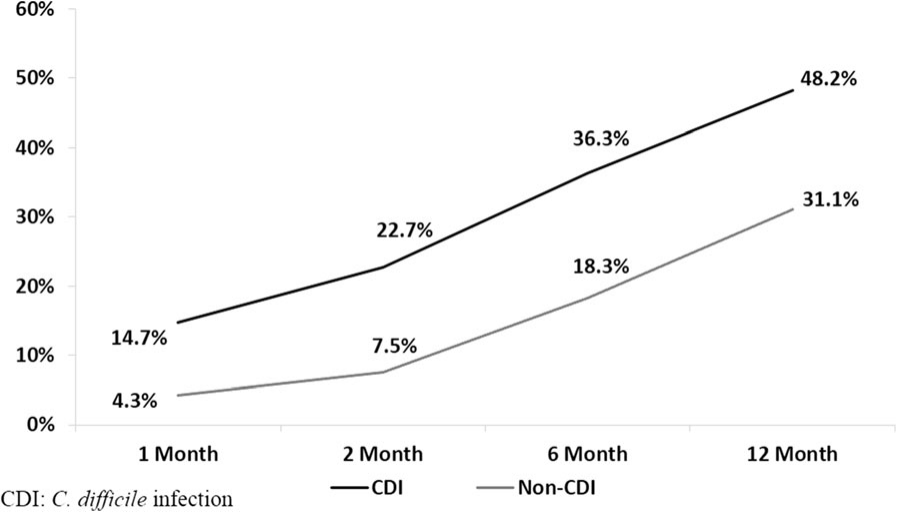
Adjusted 12-month Follow-up Mortality After the Index date Between Non-CDI and CDI Residents. CDI: *C. difficile* infection

During the 12-month follow-up period, the 30-day (14.7% vs 4.3%, *P* < 0.001) mortality rate was significantly higher among the propensity score (PS)-matched CDI residents as compared to the PS-matched non-CDI residents, so are mortality rates at 60-day, 6-month and 1-year follow up (Fig. 1).

### Health care utilization

Among Medicare-covered claims, during the 2-month follow-up period, the number of inpatient claims was significantly higher among PS-matched CDI residents compared to PS-matched non-CDI residents (0.66 vs 0.13, *P* < 0.001). Similarly, the number of skilled nursing facility (SNF) and carrier claims was significantly higher among PS-matched CDI residents. Among the Medicaid covered claims, the number of inpatient claims was significantly higher among PS-matched CDI residents as compared to PS-matched non-CDI residents (0.23 vs 0.08, *P* < 0.001); however, the number of pharmacy claims was significantly higher in the PS-matched non-CDI cohort as compared to the PS-matched CDI cohort (2.37 vs 1.74, *p* < 0.001; Table 3).

**Table 3:** Adjusted Utilization and Costs After the Initial CDI Diagnosis Between Non-CDI and CDI Residents

	Non-CDI Residents		CDI Residents			
	(*N* = 2,612)		(*N* = 653)			
	N/Mean	%/SD	N/Mean	%/SD	*P*-value	Std diff
2- months Follow-up All-cause Health Care Utilization						
Medicare: Number of Claims						
# inpatient claims	0.13	0.41	0.66	0.88	<0.001	77.03
# outpatient claims	1.39	2.27	1.57	2.62	0.1039	7.42
# SNF claims	0.33	0.80	0.85	1.31	<0.001	47.96
# HSP claims	0.15	0.57	0.20	0.58	0.06	8.29
# HHA claims	0.01	0.10	0.00	0.06	0.181	4.90
# DME claims	0.26	0.87	0.34	1.00	0.0633	8.48
# carrier claims	5.33	5.60	10.17	9.57	<0.001	61.71
# pharmacy visits among those with ≥1 pharmacy visit	7.62	7.02	5.50	6.91	<0.001	30.37
Medicaid: Number of Claims						
# inpatient claims	0.08	0.40	0.23	0.56	<0.001	31.27
# long term care claims	3.01	4.28	2.50	4.53	0.0092	11.62
# Other therapy claims	3.82	7.88	6.10	9.57	<0.001	25.95
# pharmacy visits among those with ≥1 pharmacy visit	2.37	4.67	1.74	4.23	0.0009	14.17
2- months Follow-up All-cause Health Care Costs						
Medicare:						
Overall Costs						
Inpatient stay costs	$1435	$5664	$9678	$21206	<0.001	53.1
Outpatient costs	$550	$1480	$527	$1292	0.6931	1.7
SNF costs	$1452	$5049	$4939	$9091	<0.001	47.4
HSP costs	$551	$2140	$610	$2202	0.5343	2.7
HHA costs	$22	$474	$20	$391	0.918	0.4
DME costs	$92	$412	$126	$457	0.0877	7.7
Carrier claims costs	$835	$1882	$2463	$3677	<0.001	55.7
Pharmacy visit costs among those with ≥1 pharmacy visit	$777	$914	$629	$963	0.0004	15.8
Total costs	$5716	$10120	$18993	$27290	<0.001	64.5
Medicaid:						
Overall Costs						
Inpatient stay costs	$316	$2807	$2715	$14083	<0.001	23.6
Long term care costs	$6738	$5526	$5141	$5740	<0.001	28.4
Other therapy costs	$696	$2337	$1590	$17513	0.1935	7.2
Pharmacy visit costs among those with ≥1 pharmacy visit	$178	$650	$183	$809	0.8855	0.7
Total costs	$7928	$7088	$9628	$24163	0.0757	9.5
Combined Medicare/Medicaid costs	$13644	$11362	$28621	$33532	<0.001	59.8

*CDI Clostridium difficile* infection, *DME* Durable Medical Equipment, *HHA* Home Health Agency, *HSP*, Hospital Specific Portion, *SD* standard deviation, *Std. Diff* standardized difference, *SNF* Skilled Nursing Facility

### Health care costs

For Medicare covered expenses, inpatient costs ($9,678 vs $1,435, *P* < 0.001), SNF claims ($4,939 vs $1,452, *P* < 0.001), carrier claims ($2,463 vs $835, *P* < 0.001), and total costs ($18,993 vs $5,716, *P* < 0.001) during the 2-month follow-up period were significantly higher among CDI residents compared to non-CDI residents. For Medicaid-covered expenses, inpatient stay costs ($2,715 vs $316, *P* < 0.001) were significantly higher among those with CDI during the 2-month follow-up period (Table 3). After summing all these costs from Medicare and Medicaid, inpatient stays represented the highest incremental cost increase ($10,642) followed by SNF costs. The total CDI-attributable costs equaled $14,977, and most of these incremental costs were paid by Medicare.

## Discussion

This study estimated the burden of CDI among nursing home residents. To our knowledge, this is the first study to estimate the health care costs and utilization associated with each CDI episode in this elderly population. We reported an incidence rate of 3,753 cases per 100,000 person-years (1.03 cases per 10,000 resident days), and 3,360 CDI patients per 100,000 person-years among nursing home residents. A study conducted by Kim et al. reported that the yearly incidence rate of LTC-associated CDI increased from 0.4 cases per 10,000 person-years in 2008 to 0.8 cases per 10,000 person-years in 2009 [1]. According to the results from another study conducted in 2010, the incidence rate of CDI in nursing homes was 2.3 cases per 10,000 resident days [16]. These results suggest a persistent increasing trend of CDI incidence among the nursing home residents in recent years. The emergence of new, severe, and hypervirulent epidemic strains of *C. difficile* over the past years has particularly affected older adults and played a role in the increasing CDI rates [17].

Previous literature has noted the increased incidence of CDI; however, the majority of these studies focused on acute care settings [7, 10, 18]. Besides hospitalized patients, a trend of increased CDI incidence has been observed in nursing homes and communities [8, 19]. CDI outbreaks have been reported in nursing facilities since 1986 [5]. An influx of CDI in nursing homes from acute care may lead to subsequent transmission within the facility, leading to an overall increase in nursing home CDI rates. Residents may also acquire CDI during their stay at the nursing home, adding to the increased incidence rate [1]. Several factors may have contributed to the shift from primarily a hospital-acquired infection to a nursing home-acquired infection, such as an increase in the elderly population admitted to nursing home facilities, an increase in use of multiple medications (especially antibiotics and proton pump inhibitors [PPIs]), and multiple comorbidities among the elderly population [8]. Because nursing home residents are one of the groups most vulnerable to CDI, preventative measures are crucial to effectively prevent the disease. These include additional nursing care, meticulous hand hygiene, contact precautions, appropriate use of antimicrobials and future interventions, such as vaccines. Accompanied by a high increase in the incidence rate, high mortality associated with CDI was also observed. Although there is limited data on long-term mortality, a few studies have assessed 30-day mortality [4, 19] and our 30-day mortality result (10.4%) is similar to these studies.

As the incidence of CDI continues to rise, health care costs and utilization related to CDI will have a tremendous impact on the health care system. Previous studies reported costs attributable to CDI ranging from $8,000–$30,000 that vary with coexisting conditions [20]. However to our knowledge, no study has systematically assessed healthcare costs and utilization associated with CDI in nursing homes where incidence of CDI is quickly growing. Our study is unique since it used a large data sample by linking Medicare, Medicaid, and MDS; examined total health care costs including inpatient, outpatient, SNF, and pharmacy costs; and provided an overall picture of the costs among residents in a nursing home facility. Our study included both Medicare and Medicaid covered claims and expenses. Medicare covers relatively few LTC services, such as SNF and home health care [21, 22]. Over 60% of nursing home resident care, and an even higher percentage of long-term resident care, is paid for by state Medicaid programs [23]. Thus, providing costs using both claims data offers more generalizable results, helps to better understand the true overall resource utilization, and indicates that CDI is associated with high health care costs, especially among patients residing in nursing home facilities.

A few limitations should be noted for this study. We included both Medicare and Medicaid claims data, but a certain proportion of residents may have supplemental long-term care insurance such as Medigap plans to fill the ‘gaps’ that are not covered by Medicare plans. These direct medical costs were not captured in this study. The indirect costs, such as those associated with additional nursing care, cleaning, isolation, and possible transmission to other patients, were also not accounted for in this study. Therefore, this study underestimates the true total burden of CDI among nursing home residents. Given that Medicare and Medicaid data contain administrative information from multiple inpatient and outpatient sources, under-reporting or misclassification of health outcomes of interest may occur. Since we required patients to have at least 2 MDS quarterly assessments, the sample consisted entirely of long-term stay residents. Patients who were in the nursing home for a short amount of time and were discharged were excluded from the study.

Although the Medicare, Medicaid, and MDS datasets contain uniquely integrated data, its beneficiaries are considered a vulnerable population since they are older and have a higher disease burden. Our results demonstrated an inverse distribution of CDI incidence as the residents grew older. Few previous studies have also demonstrated such an inverse trend among nursing home residents; however, no explanations have been provided [24, 25]. To verify our findings, we did a sensitivity analysis to analyze the baseline CCI score and medical costs across the age strata. We found a similar inverse trend in which residents ≥85 years residing in nursing homes had a lower comorbidity score, health care utilization, and costs prior to the incident CDI compared to patients aged 65–74 years and 75–84 years. An explanation may be that older adults (≥85 years) are more likely to reside in a nursing home facility due to comorbid conditions and frailty, and younger seniors (65–74) are more likely to reside in a nursing home facility due to poor health and may experience increased health care exposures, putting them at a higher risk for CDI.

## Conclusions

Results from our study showed 3,753 cases per 100,000 person-years, 10.4% 30-day mortality, and $15,000 in expenditures per case. With 1.4 million people residing in nursing homes in the United States, [26, 27] we estimated that approximately 53,000 annual CDI cases were associated with 5,500 deaths and $800 million in costs among this population. These numbers suggest that *C. difficile* is endemic in nursing home facilities, and more effective disease prevention and infection control measures are warranted to prevent CDI and thus improve patient outcomes and reduce overall health care costs.

## Additional file

Additional file 1: Table S1. Clinical Characteristics and Health Care Utilizations Between Non-CDI and CDI Residents Before and After PSM. **Table S1.** includes the baseline clinical characteristics such as hospitalization, antibiotic use, and heart, musculoskeletal, neurological, and psychiatric conditions between the CDI and non-CDI residents before and after propensity score matching. CDI: *Clostridium difficile* infection; CCI: Charlson Comorbidity Index; CDS: Chronic Disease Score; MDS: Minimum Data Set; SD: standard deviation; Std. Diff: standardized difference; PSM: Propensity score matching. (DOCX 34 kb)

## Abbreviations

CCI: Charlson Comorbidity Index
CDI: *Clostridium difficile* (*C. difficile*) infection
FFS: Fee-for-service
ICD-9-CM: International Classification of Diseases, 9^th^ Revision, Clinical Modification
LTC: Long-term care
MDS: Minimum Data Set
NH: Nursing home
PPIs: Proton pump inhibitors
PS: Propensity score
PSM: Propensity score matching
SAS: Statistical Analysis System
SNF: Skilled nursing facility
