# Supplementary material for: Burden of Clostridium difficile-associated disease among patients residing in nursing homes: a population-based cohort study
Source: BMC Geriatr. 2016 Nov 25;16:193. doi: 10.1186/s12877-016-0367-2 (PMC5123396; doi:10.1186/s12877-016-0367-2)
Supplement: Additional file 1: Table S1. — Clinical Characteristics and Health Care Utilizations Between Non-CDI and CDI Residents Before and After PSM. Table S1. includes the baseline clinical characteristics such as hospitalization, antibiotic use, and heart, musculoskeletal, neurological, and psychiatric conditions between the CDI and non-CDI residents before and after propensity score matching. CDI: Clostridium difficile infection; CCI: Charlson Comorbidity Index; CDS: Chronic Disease Score; MDS: Minimum Data Set; SD: standard deviation; Std. Diff: standardized difference; PSM: Propensity score matching. (DOCX 34 kb) [file 12877_2016_367_MOESM1_ESM.docx]

Additional file

Additional file 1: Table S1. Clinical Characteristics Between Non-CDI and CDI Residents Before and After PSM

|  | **Unmatched Groups** | | | | | | **1:4 PSM-Matched Groups** | | | | | |
| --- | --- | --- | --- | --- | --- | --- | --- | --- | --- | --- | --- | --- |
|  | **Non-CDI Residents** | | **CDI Residents** | | | | **Non-CDI Residents** | | **CDI Residents** | | | |
|  | **(N = 30258)** | | **(N = 789)** | | | | **(N = 2612)** | | **(N = 653 )** | | | |
|  | **N/**  **Mean** | **%** | **N/**  **Mean** | **%** | **P-value** | **Std. Diff.** | **N/**  **Mean** | **%** | **N/**  **Mean** | **%** | **P-value** | **Std. Diff.** |
| **Index** | | | | | | | | | | | | |
| **CCI Score (mean±SD)** | 3.2 | 2.4 | 4.6 | 2.8 | <0.001 | 55.2 | 4.2 | 2.7 | 4.2 | 2.6 | 0.949 | 0.3 |
| **CDS (mean±SD)** | 6.3 | 4.4 | 7.5 | 4.8 | <0.001 | 26.6 | 7.3 | 4.5 | 7.0 | 4.6 | 0.2501 | 5.1 |
| **Baseline 1 Year Hospitalization** | | | | | | | | | | | | |
| # residents with at least 1 hospitalization | 9800 | 32.4% | 511 | 64.8% | <0.001 | 68.4 | 1567 | 60.0% | 377 | 57.7% | 0.2929 | 4.6 |
| # residents with at least 2 hospitalizations | 3864 | 12.8% | 292 | 37.0% | <0.001 | 58.4 | 780 | 29.9% | 179 | 27.4% | 0.2189 | 5.4 |
| **Prior 90 Days Hospitalization** | | | | | | | | | | | | |
| # residents with at least 1 hospitalization | 3301 | 10.9% | 341 | 43.2% | <0.001 | 78.0 | 856 | 32.8% | 215 | 32.9% | 0.9406 | 0.3 |
| # residents with at least 2 hospitalizations | 646 | 2.1% | 108 | 13.7% | <0.001 | 43.8 | 180 | 6.9% | 44 | 6.7% | 0.8899 | 0.6 |
| **Antibiotic Use, 1 Year Pre-index Date** | | | | | | | | | | | | |
| Antibiotic use (yes/no) | 19766 | 65.3% | 628 | 79.6% | <0.001 | 32.3 | 2035 | 77.9% | 503 | 77.0% | 0.6286 | 2.1 |
| # days on antibiotics (mean±SD) | 24.7 | 52.8 | 32.85 | 49.2 | <0.001 | 16.0 | 33.4 | 58.1 | 31.8 | 51.0 | 0.4829 | 2.9 |
| **Antibiotic Use, 90 Days Pre-index Date** | | | | | | | | | | | | |
| Antibiotic use (yes/no) | 10256 | 33.9% | 455 | 57.7% | <0.001 | 49.1 | 1393 | 53.3% | 351 | 53.8% | 0.847 | 0.8 |
| # of days on antibiotics (mean±SD) | 5.9 | 14.3 | 10.45 | 16.4 | <0.001 | 29.8 | 9.7 | 17.5 | 9.7 | 16.3 | 0.9014 | 0.5 |
| **Gastric Acid Suppressant Use, 1 Year Pre-index Date** | | | | | | | | | | | | |
| Gastric acid suppressant use (yes/no) | 11859 | 39.2% | 395 | 50.1% | <0.001 | 22.0 | 1234 | 47.2% | 310 | 47.5% | 0.9162 | 0.5 |
| # of days on gastric acid suppressants (mean±SD) | 93.7 | 138.4 | 107.21 | 136.1 | 0.0068 | 9.8 | 104.8 | 137.6 | 104.3 | 136.3 | 0.923 | 0.4 |
| **Gastric Acid Suppressant Use, 90 days Pre-index Date** | | | | | | | | | | | | |
| Gastric acid suppressant use (yes/no) | 9738 | 32.2% | 320 | 40.6% | <0.001 | 17.5 | 1005 | 38.5% | 250 | 38.3% | 0.9283 | 0.4 |
| # of days on gastric acid suppressants (mean±SD) | 20.0 | 32.0 | 23.10 | 32.4 | 0.0077 | 9.6 | 23.1 | 33.0 | 22.4 | 32.1 | 0.6237 | 2.1 |
| **Baseline 1 Year Diseases From MDS Data** | | | | | | | | | | | | |
| **Endocrine/Metabolic/Nutritional:** | | | | | | | | | | | | |
| Diabetes mellitus | 10136 | 33.5% | 327 | 41.4% | <0.001 | 16.5 | 1014 | 38.8% | 251 | 38.4% | 0.8575 | 0.8 |
| Hyperthyroidism | 272 | 0.9% | 12 | 1.5% | 0.07 | 5.7 | 35 | 1.3% | 10 | 1.5% | 0.7075 | 1.6 |
| Hypothyroidism | 6410 | 21.2% | 172 | 21.8% | 0.6764 | 1.5 | 547 | 20.9% | 136 | 20.8% | 0.9485 | 0.3 |
| **Heart/Circulation** | | | | | | | | | | | | |
| Arteriosclerotic heart disease | 4458 | 14.7% | 126 | 16.0% | 0.3339 | 3.4 | 426 | 16.3% | 100 | 15.3% | 0.536 | 2.7 |
| Cardiac dysrhythmias | 4623 | 15.3% | 125 | 15.8% | 0.6638 | 1.6 | 439 | 16.8% | 102 | 15.6% | 0.4656 | 3.2 |
| Congestive heart failure | 7111 | 23.5% | 231 | 29.3% | 0.0002 | 13.1 | 754 | 28.9% | 182 | 27.9% | 0.6149 | 2.2 |
| Deep vein thrombosis | 707 | 2.3% | 28 | 3.5% | 0.027 | 7.2 | 89 | 3.4% | 20 | 3.1% | 0.6611 | 1.9 |
| Hypertension | 21168 | 70.0% | 589 | 74.7% | 0.0045 | 10.5 | 1963 | 75.2% | 484 | 74.1% | 0.5856 | 2.4 |
| Hypotension | 444 | 1.5% | 24 | 3.0% | 0.0003 | 10.6 | 74 | 2.8% | 16 | 2.5% | 0.593 | 2.4 |
| Peripheral vascular disease | 4928 | 16.3% | 167 | 21.2% | 0.0003 | 12.5 | 496 | 19.0% | 128 | 19.6% | 0.7218 | 1.6 |
| Other cardiovascular disease | 6886 | 22.8% | 206 | 26.1% | 0.0269 | 7.8 | 642 | 24.6% | 166 | 25.4% | 0.6555 | 1.9 |
| **Musculoskeletal** | | | | | | | | | | | | |
| Arthritis | 11139 | 36.8% | 287 | 36.4% | 0.8011 | 0.9 | 964 | 36.9% | 234 | 35.8% | 0.6112 | 2.2 |
| Hip fracture | 1350 | 4.5% | 43 | 5.4% | 0.1855 | 4.6 | 150 | 5.7% | 34 | 5.2% | 0.5953 | 2.4 |
| Missing limb (amputation) | 658 | 2.2% | 25 | 3.2% | 0.0602 | 6.2 | 72 | 2.8% | 17 | 2.6% | 0.8298 | 0.9 |
| Osteoporosis | 7492 | 24.8% | 186 | 23.6% | 0.4458 | 2.8 | 612 | 23.4% | 165 | 25.3% | 0.324 | 4.3 |
| Pathological bone fracture | 165 | 0.5% | 6 | 0.8% | 0.4202 | 2.7 | 16 | 0.6% | 5 | 0.8% | 0.6615 | 1.9 |
| **Neurological** | | | | | | | | | | | | |
| Alzheimer's disease | 6368 | 21.0% | 164 | 20.8% | 0.8597 | 0.6 | 569 | 21.8% | 142 | 21.7% | 0.9831 | 0.1 |
| Aphasia | 2126 | 7.0% | 76 | 9.6% | 0.0049 | 9.4 | 223 | 8.5% | 56 | 8.6% | 0.975 | 0.1 |
| Cerebral palsy | 198 | 0.7% | 2 | 0.3% | 0.1647 | 6.0 | 6 | 0.2% | 1 | 0.2% | 0.7052 | 1.8 |
| Cerebrovascular accident (stroke) | 7294 | 24.1% | 234 | 29.7% | 0.0003 | 12.5 | 716 | 27.4% | 171 | 26.2% | 0.529 | 2.8 |
| Dementia other than Alzheimer's disease | 13839 | 45.7% | 389 | 49.3% | 0.0472 | 7.1 | 1277 | 48.9% | 311 | 47.6% | 0.5634 | 2.5 |
| Hemiplegia/ hemiparesis | 3563 | 11.8% | 122 | 15.5% | 0.0016 | 10.8 | 371 | 14.2% | 92 | 14.1% | 0.94 | 0.3 |
| Multiple sclerosis | 263 | 0.9% | 8 | 1.0% | 0.6661 | 1.5 | 28 | 1.1% | 7 | 1.1% | 1 | 0.0 |
| Paraplegia | 141 | 0.5% | 6 | 0.8% | 0.2342 | 3.8 | 13 | 0.5% | 6 | 0.9% | 0.2057 | 5.0 |
| Parkinson's disease | 2272 | 7.5% | 55 | 7.0% | 0.5711 | 2.1 | 181 | 6.9% | 44 | 6.7% | 0.8629 | 0.8 |
| Quadriplegia | 99 | 0.3% | 4 | 0.5% | 0.3859 | 2.8 | 13 | 0.5% | 4 | 0.6% | 0.7153 | 1.5 |
| Seizure disorder | 2849 | 9.4% | 87 | 11.0% | 0.1269 | 5.3 | 292 | 11.2% | 65 | 10.0% | 0.3696 | 4.0 |
| Transient ischemic attack | 1049 | 3.5% | 32 | 4.1% | 0.373 | 3.1 | 109 | 4.2% | 29 | 4.4% | 0.7608 | 1.3 |
| Traumatic brain injury | 151 | 0.5% | 3 | 0.4% | 0.6391 | 1.8 | 13 | 0.5% | 3 | 0.5% | 0.9003 | 0.6 |
| **Psychiatric/Mood** | | | | | | | | | | | | |
| Anxiety disorder | 6530 | 21.6% | 163 | 20.7% | 0.5341 | 2.3 | 583 | 22.3% | 139 | 21.3% | 0.5692 | 2.5 |
| Depression | 18086 | 59.8% | 503 | 63.8% | 0.0244 | 8.2 | 1651 | 63.2% | 415 | 63.6% | 0.8702 | 0.7 |
| Manic depression (bipolar disease) | 1461 | 4.8% | 41 | 5.2% | 0.6344 | 1.7 | 137 | 5.2% | 37 | 5.7% | 0.6683 | 1.9 |
| Schizophrenia | 2505 | 8.3% | 48 | 6.1% | 0.0267 | 8.5 | 163 | 6.2% | 44 | 6.7% | 0.6406 | 2.0 |
| **Pulmonary** | | | | | | | | | | | | |
| Asthma | 1189 | 3.9% | 48 | 6.1% | 0.0023 | 9.9 | 140 | 5.4% | 32 | 4.9% | 0.6383 | 2.1 |
| Emphysema/ chronic obstructive pulmonary disease | 5761 | 19.0% | 194 | 24.6% | <.0001 | 13.5 | 569 | 21.8% | 150 | 23.0% | 0.5127 | 2.8 |
| **Sensory** | | | | | | | | | | | | |
| Cataract | 3833 | 12.7% | 90 | 11.4% | 0.2926 | 3.9 | 298 | 11.4% | 76 | 11.6% | 0.8691 | 0.7 |
| Diabetic retinopathy | 342 | 1.1% | 21 | 2.7% | <0.001 | 11.2 | 49 | 1.9% | 10 | 1.5% | 0.5544 | 2.7 |
| Glaucoma | 2969 | 9.8% | 99 | 12.5% | 0.011 | 8.7 | 299 | 11.4% | 75 | 11.5% | 0.9781 | 0.1 |
| Macular degeneration | 2225 | 7.4% | 51 | 6.5% | 0.3439 | 3.5 | 182 | 7.0% | 44 | 6.7% | 0.8361 | 0.9 |
| **Other**: | | | | | | | | | | | | |
| Allergies | 9317 | 30.8% | 266 | 33.7% | 0.0795 | 6.3 | 872 | 33.4% | 225 | 34.5% | 0.604 | 2.3 |
| Anemia | 8689 | 28.7% | 274 | 34.7% | 0.0002 | 12.9 | 821 | 31.4% | 215 | 32.9% | 0.4634 | 3.2 |
| Cancer | 1964 | 6.5% | 45 | 5.7% | 0.3748 | 3.3 | 145 | 5.6% | 37 | 5.7% | 0.9089 | 0.5 |
| Renal failure | 2151 | 7.1% | 88 | 11.2% | <0.001 | 14.1 | 259 | 9.9% | 65 | 10.0% | 0.9767 | 0.1 |

CDI: Clostridium difficile infection; CCI: Charlson Comorbidity Index; CDS: Chronic Disease Score; MDS: Minimum Data Set; SD: standard deviation; Std. Diff: standardized difference; PSM: Propensity score matching.
